# Screening Different Divalent and Trivalent Metals Containing Binary and Ternary Layered Double Hydroxides for Optimum Phosphate Uptake

**DOI:** 10.1038/s41598-019-52031-w

**Published:** 2019-10-29

**Authors:** Sattam Fahad Almojil, Mohamed Abdelhalim Othman

**Affiliations:** Department of Civil Engineering, King Saud University, PO Box 800, Riyadh, 11421 Saudi Arabia

**Keywords:** Pollution remediation, Chemical engineering

## Abstract

The elements constituting a layered double hydroxides material provide many alternatives for its optimization. Ten different layered double hydroxides materials with various combinations of Ni, Cu, Zn, Al, Cr, and Fe elements were studied as sorbent materials for phosphate ion. The type of element used in the layered double hydroxides affected the uptake capacity of phosphate. The influence of a specific element alone was not the primary role of enhancing the sorption performance of phosphate ion on the LDHs material. However, using specific two or three elements together is the key to achieve the best result due to synergistic effects. BET surface area of the sorbent showed no correlation with phosphate uptake. From the examined materials, Four layered double hydroxides of Cu-Zn-Cr, Zn-Cr, Ni-Al, and Cu-Ni-Cr showed high phosphate sorption capability. Sorption equilibrium isotherm, reaction kinetics, and desorption of phosphate from the sorbent materials were also investigated.

## Introduction

Pollution of water resources hinders access to clean water to meet the needs of the continuously growing world population. Phosphate resulting from agricultural runoffs, domestic sewage, and through the sewage generated from mining activities, is a major pollutant of water bodies. Phosphate creates significant problems in aquatic environments; because as a fertilizer, it causes the growth of algae and microorganisms known as eutrophication, leading to the depletion of dissolved oxygen and causing deterioration in the aquatic environment and water quality. Several studies have investigated methods to remove phosphorus from aqueous solutions; these include enhanced biological phosphorus adsorption, chemical precipitation, crystallization, ion exchange, and adsorption^1,2^. Among these methods, adsorption is an effective and promising technology regarding cost and ease of operation^3,4^.

Layered double hydroxides (LDHs) are a class of anionic clays that show high anion exchange capacity, making it a promising adsorbent material in many industrial applications. LDHs have been widely used as an adsorbent, ion exchange, or a catalyst material to remove organic and inorganic impurities from polluted water^5–8^. The structure of LDHs contains positively charged layers due to the presence of two metal hydroxides in different oxidation states, which retained its charge-balance by the presence of anions in the interlayer region^9^. Different studies that used LDHs for phosphate removal and recovery showed promising results^10,11^. However, most of these studies focused on using LDHs that consist of Mg and Al elements in their structure without investigating the possibilities of using different elements^11–16^. Other studies replaced Mg and Al elements with Zn and Fe^17–21^. LDHs can provide more options for elements that can be used for their synthesis^9^, which opens the door for tuning this material for phosphate removal and recovery from aqueous solution. Different variables can be utilized in the synthesis of LDHs to produce materials specifically designed for the required application. Adjusting LDHs is a critical factor for the preparation of high-performance sorption materials^9,22^. The refinement can be accomplished by altering of the metal cations, changing the proportions between divalent and trivalent ions, accommodating an appropriate anion into the interlayer space, controlling thermal activation, and stimulants by doping with different elements. A screening study for phosphate sorption using thirteen different LDHs and related structure shows a Zn-Fe-Zr combination with high potential for phosphate uptakes^23^.

Many natural copper deposits contain some amount of phosphate ion in its structure, like Turquoise, Coeruleolactite, Wooldridgeite, Cloncurryite, and Hentschelite mineral. Although copper may harm the environment, the tendency of copper elements to attract phosphate ions make it a potential ingredient candidate for the preparation of LDHs, whereas no previous study examines this possibility.

While LDHs naturally exist as mineral deposits, they are feasible and economically produced. Two different metal hydroxides with different oxidation state forming the layers of LDHs material produces a positive charge between these layers^24^. Balancing negatively charged anions resides the interlayer space to sustain its integrity. The general formula of the LDHs is expressed as [M_1−*x*_^(II)^ M_*x*_^(III)^ (OH)_2_] A_*x/n*_^*n*−^ *m*H_2_O. Where A^n−^ is the anion occupying the interlayers space with water molecules, M^(II)^ and M^(III)^ is the divalent and trivalent metal ion, respectively. The primary mechanism that allows the sorption of anions by LDHs is the anion exchange with the interlayers LDHs’ anion^24–26^.

The objective of this study was to screen a different combination of divalent and trivalent ions incorporated in the LDHs structure for the best sorption removal of phosphate. Divalent ions consisting of Ni, Cu, Zn, and trivalent ions consisting of Al, Cr, and Fe were used to prepare the LDHs material, which gave a different combination of LDHs materials. The effects of some factors, such as time, initial phosphate concentration, and pH, were investigated. The composition of the LDHs sorbents was confirmed using inductively coupled plasma optical emission spectrometer, and the morphological structure was examined with X-ray diffraction.

## Experimental

### Preparation of sorbent materials

Different preparation routes for LDHs have been described in the literature; the most straightforward and widely used methods are coprecipitation^25,27^. In this method, solutions of divalent and trivalent elements containing the anion that is to be incorporated into the LDHs are used as precursors. The synthesis is performed under a condition of supersaturation, to ensure simultaneous precipitation of the two cations. Metals nitrates were used for making the first metal solutions of M^(II)^ and M^(III)^. Caustic soda was used as a base solution and was related to the metal solution according to the following factor, [NaOH] = 1.6 [M^(II)^ + M^(III)^], with the interlayer anion of carbonate, [CO_3_^2−^] = 2.0 [M^(III)^]. The metals solution and the base solution were mixed concurrently for 2 min; then the formed mixture was aged at 85 °C for 24 h. The precipitates were washed with distilled water to remove the remaining salts and then dried at 100 °C for 24 h^28^. The final solids were ground and passed through 0.25 mm sieve, then stored for later use. Obtaining pure LDHs was indicated by limiting the ratio M^(II)^/M^(III)^ between 2 and 4^24,25^. The exchange capacity of the LDHs could be increased by increasing the amount of the trivalent cations in the LDHs, which consequently increase the net positive charge in the interlayer region^24^. In this study, the ratio M(II)/M(III) was chosen to equal two to increase the amount of the trivalent cations and hence the anion exchange capacity.

### Characterization of prepared sorbents

A sample of prepared material was digested by nitric acid, using a 1:1 weight ratio of nitric acid. The solution was heated for two hours at 90 °C to dissolve the metals. The concentration of metals was measured using an inductively coupled plasma optical emission spectrometer (ICP-OES, Varian 730-ES). An X-ray diffraction patterns were obtained using an X-ray diffractometer (Bruker-D8 Discover) equipped with Cu Kα radiation (λ = 1.5406 Å). Data were recorded over a 2θ range of 5°–70° with a step size of 0.02°. The test was run on a standard glass slide for the background correction. The specific surface area for the prepared samples was measured using a Micrometrics Tristar II surface area analyzer, by N_2_ adsorption, whereas degassing of the samples were conducted at 80 °C for each 0.3 g of the LDHs material. The concentration of phosphate ions in the solution phase was determined using the standard procedure of vanadomolybdo-phosphoric acid colorimetric method^29^ and a portable Spectrophotometer (HACH DR/3000).

### Preparation of aqueous phosphate solution

A stock solution of phosphate (P), containing 1000 mg P/L, was prepared by dissolving potassium dihydrogen orthophosphate (KH_2_PO_4_) powder in distilled water. Working solutions of phosphate with different concentrations were prepared by diluting the stock solution with distilled water. The working solutions were used for batch sorption test with a pH of about 6.0 except for sorption test of pH-effects.

### Procedural specification for batch sorption experiment

Firstly, for screening the best LDHs material for phosphate uptakes, a sample size of 200 mg of LDHs material was used. Then, 100 mL of phosphate solution containing 20 mg P/L was added to an Erlenmeyer flask and mixed with the LDHs. The experiments were performed at pH = 6 to approximate the real condition of pH neutrality. The flask was covered and placed on a shaker at 150 rpm for 24 h at room temperature of 23 °C, to ensure the equilibrium of phosphate concentration between the liquid phase and the solid phase. After the 24 h period, the suspension was filtered through 1.6-μm borosilicate glass microfiber filters and then analyzed for phosphate concentration. Secondly, for sorption isotherm, sorption kinetics, pH effects, and phosphate desorption performance, the detail of experimentation characteristics are shown in Table 1.

**Table 1:** Specification of batch sorption experiment.

Experiment	Sample size (mg of LDHs)	Volume of solution (mL)	Initial phosphate concentration (mg/L)	Duration of the sorption process
Screening best LDHs material	200	100	20	24 h
Sorption isotherm	50	50	5, 10, 20, 40, 80, 100 and 160	24 h
Sorption kinetics	700	700	20	5, 10, 15, 20, 30, 45, 60, 90 and 120 min
pH effects	50	50	20	24 h
Desorption performance	400	400	20	24 h

### Measurement of phosphate uptake

Mass balances, as expressed in Eq. (1), were used to calculate the mass of phosphate adsorbed per unit mass of the LDHs material after equilibrium (*Q*_*e*_, mg P/g). *V* is the volume of solution (L); *C*_*i*_ and *C*_*e*_ are the initial and equilibrium concentration of phosphate in solution after adsorption process (mg/L), respectively; and *m* is the LDHs mass (g).

1 $${\rm{Adsorption}}\,{\rm{quantity}},\,\,{Q}_{e}=\frac{V({C}_{i}-{C}_{e})}{m}$$

The affinity of dissolved phosphate to the LDHs material was evaluated using Freundlich and Langmuir isotherm models described in Eqs (2) and (3), respectively. K_f_ and K_L_ represent the Freundlich isotherm capacity parameter ((mg/g)(L/mg)^1/n^) and the Langmuir bonding term related to interaction energies (L/mg), respectively; 1/n is the Freundlich isotherm intensity parameter (unitless); q_max_ denotes the Langmuir maximum capacity (mg/g).

2 $${\rm{Freundlich}}\,{\rm{isotherm}},\,\,{Q}_{e}={K}_{f}{C}_{e}^{\frac{1}{n}}$$

3 $${\rm{Langmuir}}\,{\rm{isotherm}},\,\,{Q}_{e}=\frac{{q}_{max}{K}_{L}{C}_{e}}{1+{K}_{L}{C}_{e}}$$

Pseudo-first-order and pseudo-second-order kinetic models^30^ as expressed in Eqs (4) and (5), respectively, were used to estimate the rate of adsorption of phosphate on the LDHs material. *Q*_*t*_ is the mass of phosphate adsorbed per unit mass of LDHs at time *t* (mg/g); *k*_1_ and *k*_2_ are the first-order and second-order reaction rate constants (1/h and g/mg/h), respectively.

4 $$\mathrm{Pseudo} \mbox{-} \mathrm{first} \mbox{-} \mathrm{order}\,\,{Q}_{t}={Q}_{e}\,(1-{e}^{-{k}_{1}t})$$

5 $$\mathrm{Pseudo} \mbox{-} \mathrm{second} \mbox{-} \mathrm{order}\,\,{Q}_{t}=\frac{{Q}_{e}^{2}{k}_{2}t}{(1+{Q}_{e}{k}_{2}t)}$$

### Effects of pH and desorption study

The effect of pH on phosphate uptake was investigated at different pH values using HCl or NaOH solution, which reflect a range of acidic and basic solutions; Table 1 describes the conditions of the experiment. Desorption study was conducted for the exhausted LDHs material, whereas the LDHs was subjected to sorption with an initial phosphate concentration of 20 mg P/L using 400 mg of sorbent, more detail in Table 1. Two desorption solutions were used, a solution of nitric acid with pH = 4, and a solution of sodium hydroxide, as alkaline, with pH = 12.

## Result and Discussion

### Characteristics of prepared sorbents

**Figure 1:**
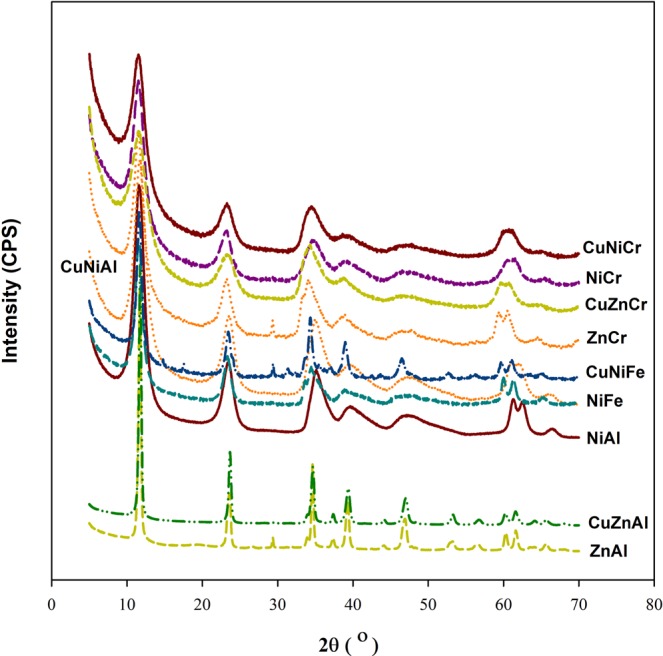
XRD patterns for the synthesized LDHs sorbents.

Combining the divalent ions of Ni, Cu, Zn with the trivalent ions of Al, Cr, Fe results in nine LDHs materials. However, based on Reichle^31^ study, Cu-LDHs require an additional divalent ion along with Cu to obtain the desired structure of LDHs. Accordingly, twelve different sorbent materials were formed (see Supplementary Fig. S1). However, after characterizing the synthesized material by XRD, Zn-Fe, and Cu-Zn-Fe compounds failed to acquire the morphology structure of the LDHs material. Figure 1 shows the XRD for the other ten LDHs materials. The type of synthesized LDHs along with their ICP-OES chemical composition and other characteristics are presented in Table 2. A good match between the chemical composition of the nominal and as-synthesized LDHs material was achieved. High variation of BET surface area for the prepared LDHs material had been observed, which could influence the sorption capacity of the LDHs; Cu-Zn-Cr compound showed the highest surface area that can be elucidated due to the formation of the smallest nanoparticle sized compared to other LDHs material. The attribute of the smallest particle size for Cu-Zn-Cr LDHs could be explained by the synergetic effect of these metals when used as precursors in the formation of the LDHs utilizing the coprecipitation method, more investigation is required.

**Table 2:** Characteristics of sorbents and phosphate uptake quantity in the screening experiment.

SN	Composition of sorbent^(a)^ (Mole ratio of elements)		BET surface area (m^2^/g)	Pore Volume^(c)^ (cm^3^/g)	Nanoparticle size^(c)^ (nm)	Phosphate Sorption Quantity, mg P/g
	Nominal	As-synthesized^(b)^				
1	Ni_0.67_Al_0.33_	Ni_0.66_Al_0.34_	94	0.010	64	9.6
2	Ni_0.67_Cr_0.33_	Ni_0.66_Cr_0.34_	20	0.013	303	5.0
3	Ni_0.67_Fe_0.33_	Ni_0.64_Fe_0.36_	99	0.205	61	6.9
4	Cu_0.33_Ni_0.33_Al_0.33_	Cu_0.35_Ni_0.32_Al_0.33_	15	0.011	667	3.5
5	Cu_0.33_Ni_0.33_Cr_0.33_	Cu_0.35_Ni_0.31_Cr_0.34_	52	0.059	115	9.5
6	Cu_0.33_Ni_0.33_Fe_0.33_	Cu_0.35_Ni_0.31_Fe_0.34_	71	0.223	85	7.6
7	Cu_0.33_Zn_0.33_Al_0.33_	Cu_0.36_Zn_0.32_Al_0.32_	33	0.230	181	5.3
8	Cu_0.33_Zn_0.33_Cr_0.33_	Cu_0.33_Zn_0.32_Cr_0.35_	141	0.268	42	9.8
9	Zn_0.67_Al_0.33_	Zn_0.66_Al_0.34_	33	0.231	183	6.6
10	Zn_0.67_Cr_0.33_	Zn_0.66_Cr_0.34_	48	0.089	125	9.5

^a^All chemical formula contains the following suffix $\sim {({\rm{OH}})}_{2}{({{\rm{CO}}}_{3}^{2-})}_{0.17}\cdot {{\rm{mH}}}_{2}{\rm{O}}$.

^b^From ICP-OES chemical analysis.

^c^From N_2_ sorption measurement, BET method at 80 °C.

### Screening of phosphate uptake by different adsorbents

**Figure 2:**
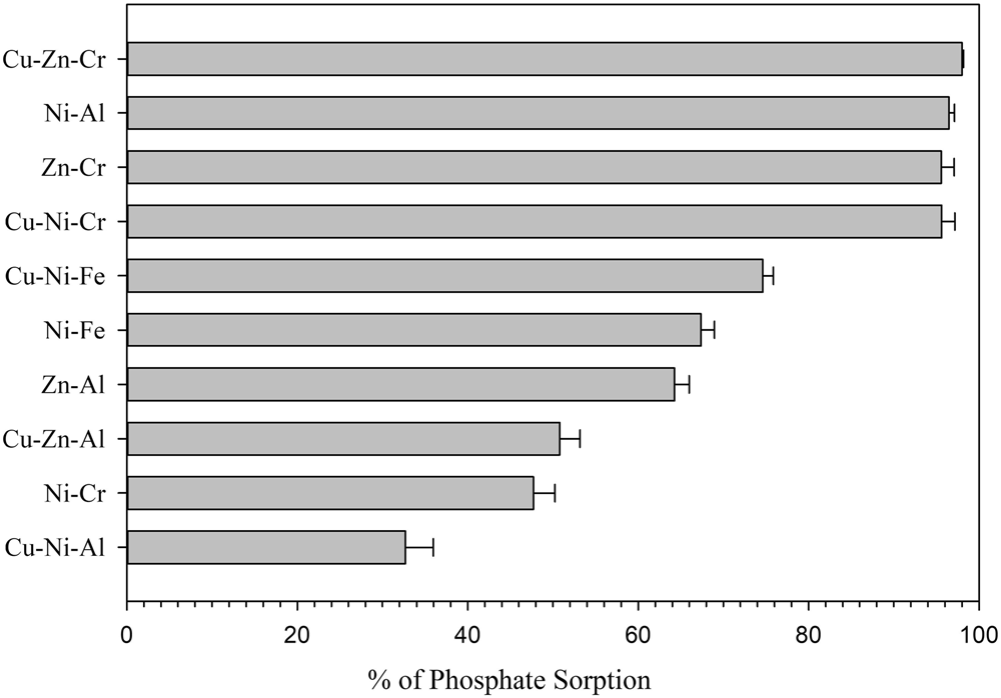
Phosphate uptakes by the different LDHs compounds.

The results of phosphate removal by the different LDHs indicate the influence of the type of metal ions incorporated into the structure of the sorbent. Cu-Zn-Cr, Zn-Cr, Ni-Al, and Cu-Ni-Cr LDHs material showed higher sorption uptake of phosphate, Fig. 2. The chemical formula for these compounds can be written based on the general formula of LDHs described previously along with as-synthesized material shown in Table 2, i.e., ${{\rm{Cu}}}_{0.33}{{\rm{Zn}}}_{0.32}{{\rm{Cr}}}_{0.35}{({\rm{OH}})}_{2}{({{\rm{CO}}}_{3}^{2-})}_{0.17}\cdot {{\rm{mH}}}_{2}{\rm{O}}$. The nature of LDHs that consists of a layered structure of positive charge facilitate the attraction of the phosphate ion. Whereas there is always a competing anion for the interlayer site, the type of metals and their proportion in the LDHs structure play a key role in attracting the phosphate ion. Furthermore, the synergistic effect of the elements may influence the improvement of phosphate ion sorption. These synergistic effects could be observed when comparing the three LDHs of Ni-Al, Zn-Al, and Zn-Cr. While Zn-Al achieved less sorption of phosphate, Ni-Al and Zn-Cr achieved high sorption. The results in Table 2 and Fig. 2 reveal that the BET surface area had less influence than the type and composition of the sorbent, which implies a chemisorption mechanism had taken place in the sorption process of phosphate to the LDHs material.

**Figure 3:**
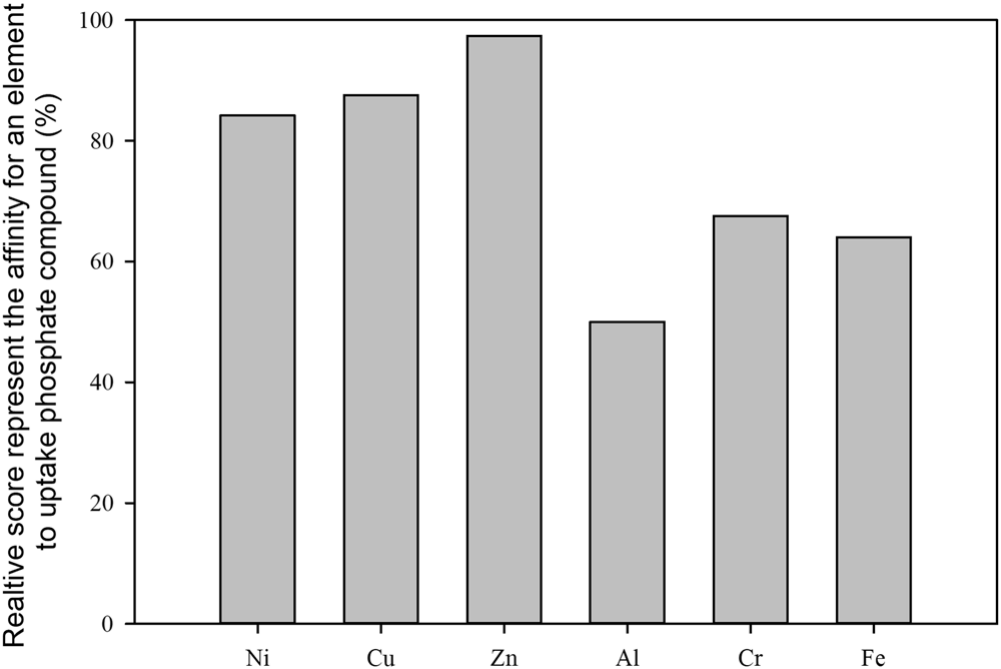
Relative affinity of elements to uptake phosphate ion.

LDHs metal ions showed a different affinity to adsorb phosphate. For the divalent ions, the affinity to phosphate has the following order Zn > Cu > Ni; and for the trivalent ions, the affinity order is Cr > Fe > Al as demonstrated in Fig. 3. The aluminum roles is mainly a structural aspect that guarantees the formation of the layered structure for LDHs material as observed by a previous study^28^. Of these four enhanced LDHs compounds, Cu-Zn-Cr LDHs was selected for more kinetics and sorption isotherm studies, as it achieves the higher phosphate uptakes.

### Phosphate sorption isotherm

**Figure 4:**
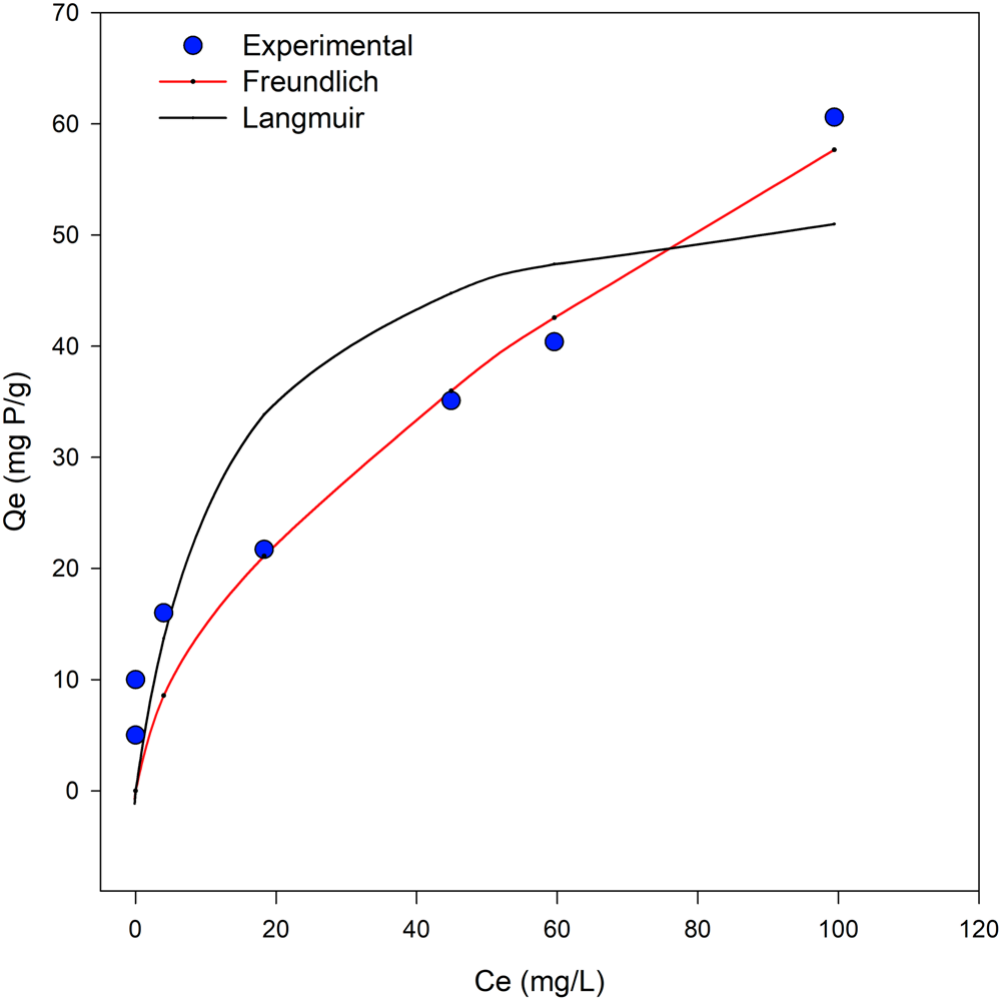
Freundlich and Langmuir sorption isotherm of phosphate onto Cu-Zn-Cr LDHs.

The results of phosphate sorption isotherm for Cu-Zn-Cr LDHs are shown in Fig. 4, where experimental data was compared to Freundlich and Langmuir isotherm, Eqs (2) and (3) respectively. The experimental data reveal that for an initial concentration of phosphate in solution (*C*_*i*_) up to 15–20 mg/L, the sorbent was able to uptake all phosphate from solution, whereas the phosphates concentration after equilibrium was below 0.1 mg/L. Then, gradually with initial phosphate increased, the equilibrium concentration of phosphate (*C*_*e*_) demonstrated a linearity relation with the quantity of sorbed phosphate in the LDHs material (*Q*_*e*_). As shown in Fig. 4, at higher values of the equilibrium concentration of phosphate, the Freundlich model provided a better fit to the experimental data; whereas, the Langmuir model was better at lower values of initial concentrations.

### Phosphate sorption kinetics

**Figure 5:**
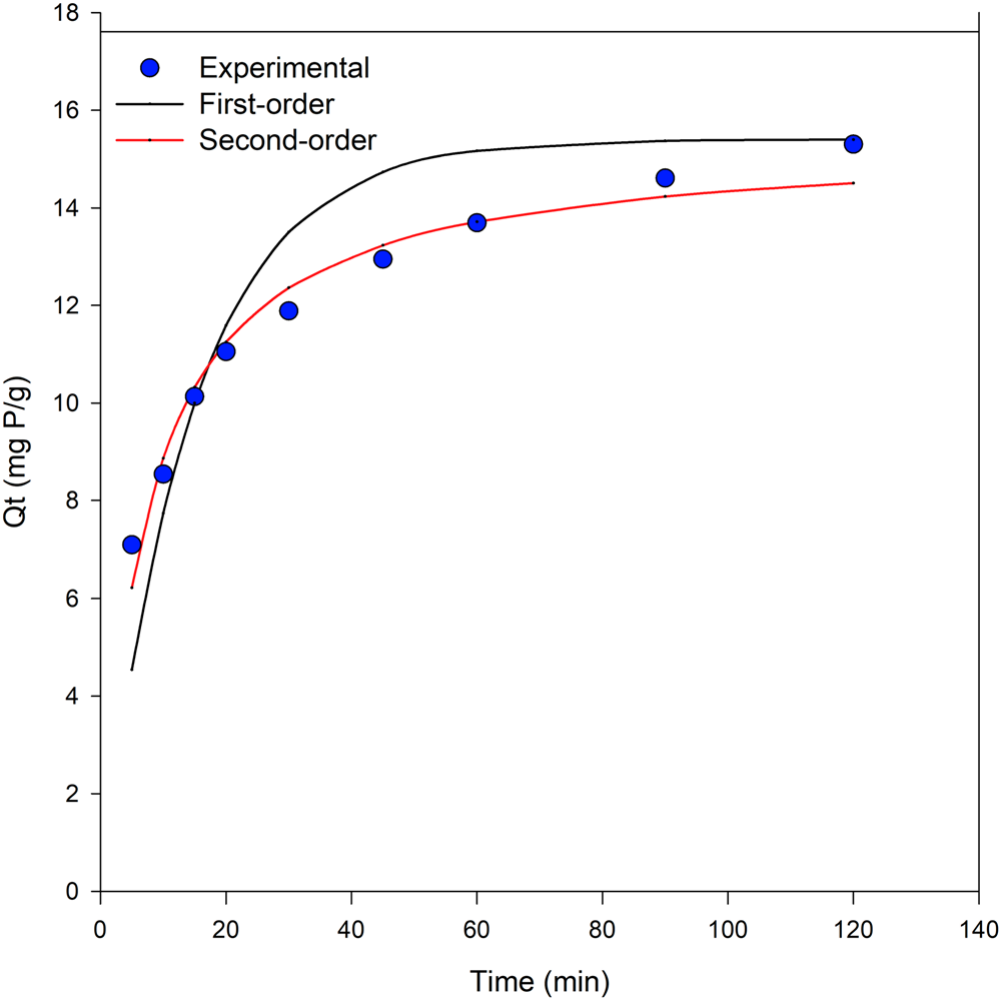
Kinetics of phosphate sorption onto Cu-Zn-Cr LDHs.

The dependence of the adsorption rate on the concentration of the adsorbate is of particular interest. The phosphate sorption by the Cu-Zn-Cr LDHs as a function of time is shown in Fig. 5. The sorption rate was relatively faster in the first 20 min, and then gradually increased until it reached an almost stable value after 100–120 min due to saturation of adsorbent. As demonstrated from the figure, the pseudo-second-order reaction fitted well the experimental data compared to pseudo-first-order; with the reaction rate constant *k*_2_ = 0.53 g/mg/h and *k*_1_ = 4.13/h, respectively. This result indicates the inclination of the reaction towards chemisorption instead of physisorption reaction reflecting the influence of the type of element selected on the performance of the sorbent material.

The superiority Cu-Zn-Cr over the other LDHs compounds can be readily correlated to HSAB principle^32^. According to the HSAB concept, Cr^3+^ has high hardness acidity (high positive charge and low polarization) compared to Al^3+^ and Fe^3+^, which is more suited to adsorb hard bases like phosphate. The presence of Cr^3+^ in the LDHs compound shows an advantage over the other ions, excluding Ni-Cr, whereas the effect of the BET surface area lessens its sorption capacity.

### Effect of pH on phosphate uptake

**Figure 6:**
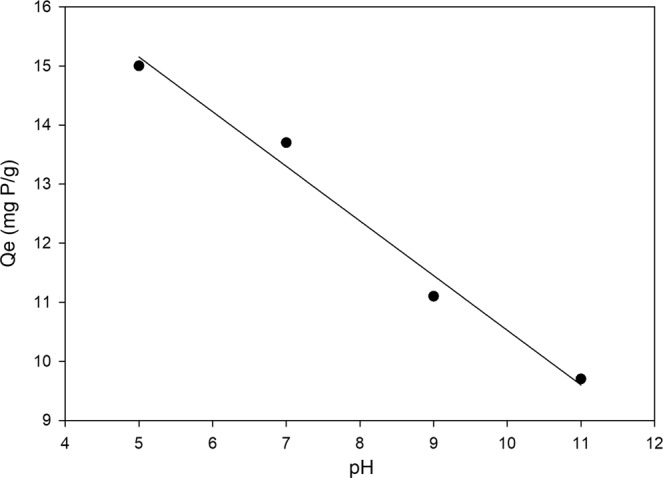
Effect of pH on the sorption capacity of phosphate onto Cu-Zn-Cr LDHs.

The Cu-Zn-Cr LDHs material was investigated for its performance to uptake phosphate for different pH values of the solution. As expected from a previous study^33^, the lower the pH value of the solution, the more capacity of LDHs sorbent material to uptake phosphate. The sorption capacity versus pH values of the solution is shown in Fig. 6, the phosphate uptake at pH = 5 is 50% greater than at pH = 11. This advantages of low pH solution were explained well by Chitrakar *et al*.^34^, as the OH^−^ ion increases the surface charge will be more negative which will interfere and decrease the anion exchange capacity of LDHs material.

### Desorption study

The exhausted Cu-Zn-Cr LDHs sorption material was tested for desorption of phosphate ion, whereas acidic and alkaline solutions were used. The alkaline solution desorbed 83% of the phosphate from the LDHs material, whereas the acidic solution desorbed only 39%. This result could be related to the incorporation of the hydroxides ions in the structure of the freshly prepared LDHs that gave the bases characteristic of this material^35^. The concentration of the Cu, Zn, and Cr metal ions were analyzed for the exhaust alkaline solution used for desorption study, whereas it was found 28.4 ± 2.5, 2.4 ± 0.2, 0.9 ± 0.1 mg/L, respectively. The copper ion seems to be likely to desorb with using an alkaline solution like NaOH, which contaminates the water and degrade the sorption material. However, as many methods could be used to remove copper ions contamination from water, the requirement for further treatment could render the recovery of phosphate ion economically infeasible. In this sense, the use of Zn-Cr LDHs material will be more justifiable where it shows excellent performance for phosphate sorption with stable Zn and Cr ions.

## Conclusion

Incorporating different type of ions in the LDHs structure leads to a versatile sorbent material with better characteristics that target specific compounds. Phosphate sorption on LDHs of a different composition was studied in a batch system. Divalent ions of Ni, Cu, Zn and trivalent ions of Al, Cr, and Fe were considered as potential candidates to synthesize LDHs for better sorption and uptake of phosphate. Using Cu, Zn, and Cr in the LDHs structure showed the best performance of sorption of phosphate from its aqueous solution. The synergy between these three ions improved the sorption of phosphate and thus its separation from water bodies. However, due to contamination of the recovery solution, that used to regenerate the sorption material with copper, the copper ion is proposed to be eliminated, and the adopting of LDHs material consist of Zn-Cr will be preferable.

Freundlich isotherm showed the best fit of the affinity of dissolved phosphate to Cu-Zn-Cr LDHs, and a pseudo-second-order reaction rate expression best described the experimental data. Chemisorption process was concluded as the type of bonding between the phosphate and the LDHs material, whereas the BET area of the sorbent has less influence on phosphate uptake.

## Supplementary information

: LDHs materials used as a sorbent for phosphate ion
