# Supplementary material for: Screening Different Divalent and Trivalent Metals Containing Binary and Ternary Layered Double Hydroxides for Optimum Phosphate Uptake
Source: Sci Rep. 2019 Oct 29;9:15511. doi: 10.1038/s41598-019-52031-w (PMC6820524; doi:10.1038/s41598-019-52031-w)
Supplement: Supplementary file 1 — : LDHs materials used as a sorbent for phosphate ion [file 41598_2019_52031_MOESM1_ESM.docx]

Supplementary Information for:

**Screening Different Divalent and Trivalent Metals Containing Binary and Ternary Layered Double Hydroxides for Optimum Phosphate Uptake**

Sattam Fahad Almojil^1^, Mohamed Abdelhalim Othman^1,*^

^1^ Department of Civil Engineering, King Saud University, PO Box 800, Riyadh 11421, Saudi Arabia

^*^ Corresponding author, email: [maothman@ksu.edu.sa](mailto:maothman@ksu.edu.sa)


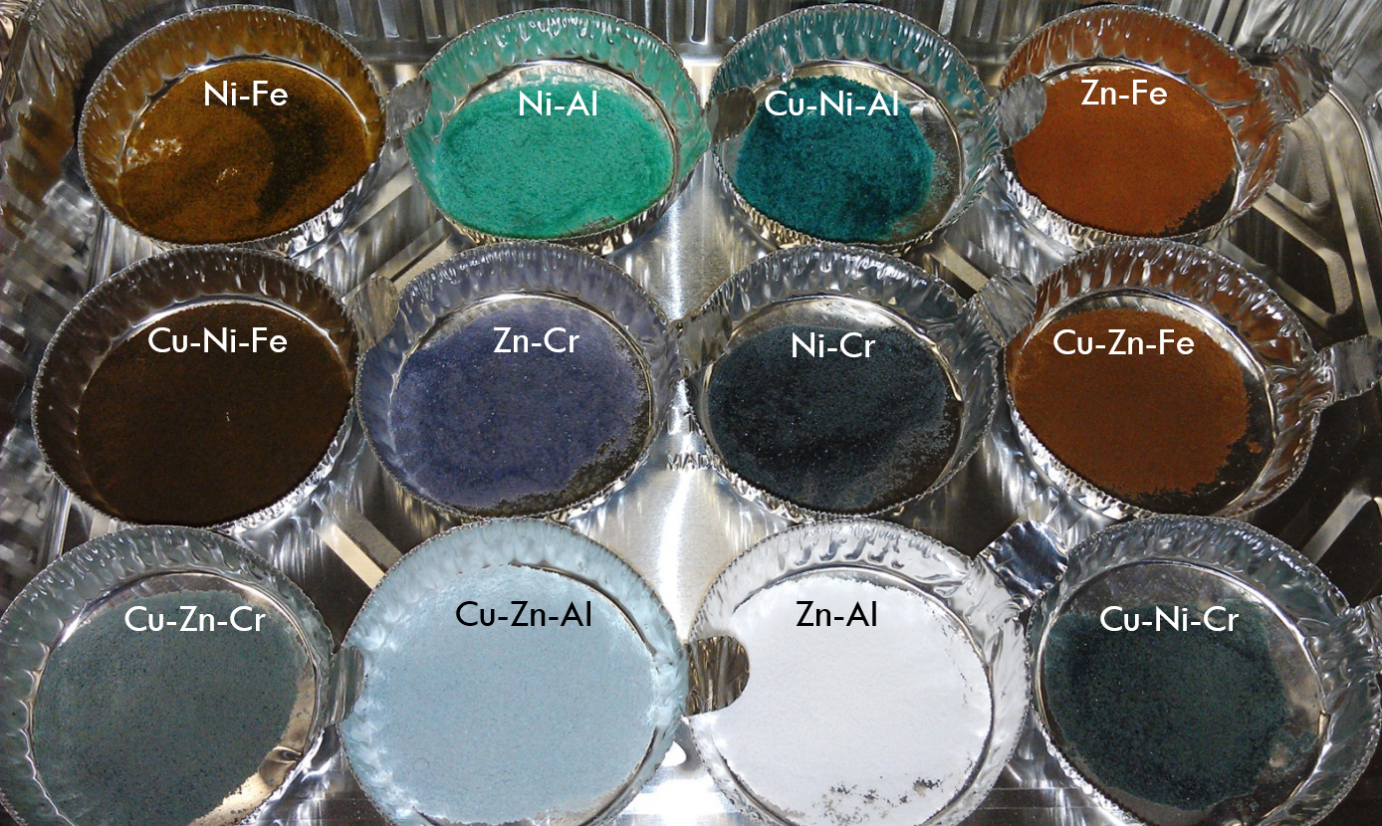


**Supplementary Figure S1**: LDHs materials used as a sorbent for phosphate ion
